# Piperacillin reaches high concentrations in bile and target tissues of the biliary system: an experimental study in pigs

**DOI:** 10.1128/aac.00792-25

**Published:** 2025-08-18

**Authors:** Louise L. Pontoppidan, Mats Bue, Kim C. Houlind, Anders R. Knudsen, Jan B. Pedersen, Magnus A. Hvistendahl, Pelle Hanberg

**Affiliations:** 1 Department of Surgery, Lillebaelt Hospital, Kolding, Denmark; 2 Department of Regional Health Research, University of Southern Denmark 6174, Odense, Denmark; 3 Department of Orthopedic Surgery, Aarhus University Hospital 11297, Aarhus, Denmark; 4 Aarhus Denmark Microdialysis Research Group (ADMIRE), Aarhus University Hospital 11297, Aarhus, Denmark; 5 Department of Vascular Surgery, Lillebaelt Hospital, Kolding, Denmark; 6 Department of Surgery, Aarhus University Hospital 11297, Aarhus, Denmark; 7 Department of Surgery, Aalborg University Hospital 53141, Aalborg, Denmark; 8 Department of Otorhinolaryngology, Head and Neck Surgery, Aarhus University Hospital 11297, Aarhus, Denmark; Providence Portland Medical Center, Portland, Oregon, USA

**Keywords:** piperacillin/tazobactam, pharmacokinetics, biliary system, microdialysis, animal model

## Abstract

The antibiotic combination of piperacillin/tazobactam (TZP) is commonly utilized for preventing and treating infections in the biliary system, with piperacillin being the primary agent. Its effectiveness is closely related to the time with concentrations above the minimal inhibitory concentration (*T* > MIC) of the bacteria involved. The most frequently encountered bacteria associated with biliary system infections present with clinical breakpoint MIC values of 8 and 16 µg/mL. This porcine study aimed to apply microdialysis to assess target site piperacillin *T* > MIC 8, 16, and 32 (4× MIC 8) µg/mL in the biliary system. In eight healthy pigs (Danish Landrace breed, weight 78–82 kg), five microdialysis catheters were placed for sampling of piperacillin concentrations in the liver, the wall of the gallbladder, the bile in the gallbladder, the wall of the common bile duct (CBD), and the bile in the CBD. A bolus of TZP 4/0.5 g was administered intravenously, and microdialysates and blood samples were collected during an 8 h period. Ultrahigh performance liquid chromatography (UHPLC) was used to quantify the piperacillin concentrations. The mean *T* > MIC (%T > MIC) varied from 345 to 446 min (77%–99%), 261–446 min (58%–99%), and 200–444 min (42%–99%) for the MIC targets of 8, 16, and 32 µg/mL, respectively. The pharmacokinetic parameters in the bile were found to be different compared to the remaining compartments with higher AUC_0–8h_ and *C*_max_ values and longer *T*_1/2_. Piperacillin displayed prolonged *T* > MIC in bile compared to plasma and the target tissues of the biliary system, approaching 100%*T* > MIC for the MIC targets of 8, 16, and 32 µg/mL.

## INTRODUCTION

Infections of the biliary system predominantly occur following surgical interventions or as a result of obstruction of the biliary tract caused by bile stones or tumors (1). Antibiotics play a key role in both preventing and treating these infections. In the last decade, correlating antibiotic concentrations with pharmacokinetic/pharmacodynamic (PK/PD) indices and the sensitivity of relevant bacteria has improved both prophylactic and treatment regimens (2).

Current international recognized guidelines recommend piperacillin/tazobactam (TZP) for both prevention and treatment of infections in the biliary system (3, 4). TZP primarily derives its efficacy from piperacillin, a β-lactam antibiotic, with efficacy best correlated with the PK/PD index of time above the minimum inhibitory concentration (*T* > MIC) of the causative bacteria (5). Previous studies have indicated that piperacillin achieves high peak drug concentrations in the bile and biliary tissue relative to plasma, exceeding the MICs of the most common bacterial pathogens, but fails to relate the findings to T > MIC (6, 7).

For surgical prophylaxis, recommendations state maintaining target tissue concentrations above a given MIC value throughout the procedure as a minimum (8). In treatment settings, it is recommended that target site concentrations of β-lactams remain above relevant MIC targets for a minimum of 50% of the dosing interval (50%*T* > MIC) (2). However, in the case of critically ill patients, a target of 100%*T* > MIC or even 100%*T* > 4× MIC has been suggested to ensure successful treatment outcomes and to minimize the risk of antimicrobial resistance development (9).

TZP offers broad-spectrum coverage against the most common infectious bacteria in the biliary system, such as *Escherichia coli*, *Klebsiella* spp., *Pseudomonas* spp., and *Enterococcus* spp (4, 10, 11). These bacteria present with MICs corresponding to the epidemiological cut-off values of 8 and 16 µg/mL, as defined by the European Committee on Antimicrobial Susceptibility Testing (12). Therefore, relating the TZP concentrations in the biliary system to the given PK/PD indices with MIC values of 8, 16, and 32 (4× MIC 8) µg/mL may potentially enhance current prophylactic and treatment regimens. Obtaining this knowledge requires dynamic sampling, which is challenging due to the anatomical location of the biliary system. To address this, we used microdialysis in a porcine model. The primary aim of this study was to determine piperacillin *T* > MIC in bile and the target tissues of the biliary system in relation to MIC targets of 8, 16, and 32 µg/mL. Secondly, it is to relate the *T* > MIC to the treatment targets of 50 and 100%T > MIC of 8, 16, and 32 µg/mL.

## MATERIALS AND METHODS

### Study design

In this prospective observational study, microdialysis catheters were placed in the biliary system in pigs for a dynamic 8 h quantification of piperacillin concentrations. The study was carried out at the Institute of Clinical Medicine at Aarhus University Hospital, Denmark. All chemical analyzes were performed by BioXpedia A/S, Aarhus, Denmark.

### Animals, anesthetics, and monitoring

Eight female pigs (Danish Landrace breed) weighing 78–82 kg were included. Before arriving at the operating room, the pigs were sedated with Zoletil mix ([25 mg/mL tiletamin + 25 mg/mL zolazepam] + 6.25 mL xylazine [20 mg/mL] + 1.25 mL ketamine [100 mg/mL] + 2.5 mL butorphanol [10 mg/mL], 1 mL/10 kg). Upon arrival, the pigs were intubated, and urine, peripheral, central venous, and arterial catheters were placed. Vital signs (blood pressure, heart rate, oxygen saturation, and body temperature) were monitored throughout the study. To maintain a body temperature of approximately 37°C, blankets and ice packs were used to regulate the temperature. Arterial pH was kept within 7.35–7.45 and regulated by ventilation. Propofol (Fresenius Kabi, Bad Homburg, Germany) (550 mg/h, continuous infusion) and fentanyl (B. Braun, Melsungen, Germany) (0.6 mg/h, continuous infusion) were used to keep the pigs anesthetized. At the end of the study, the pigs were euthanized with pentobarbital.

### The surgical procedure

**Fig 1:**
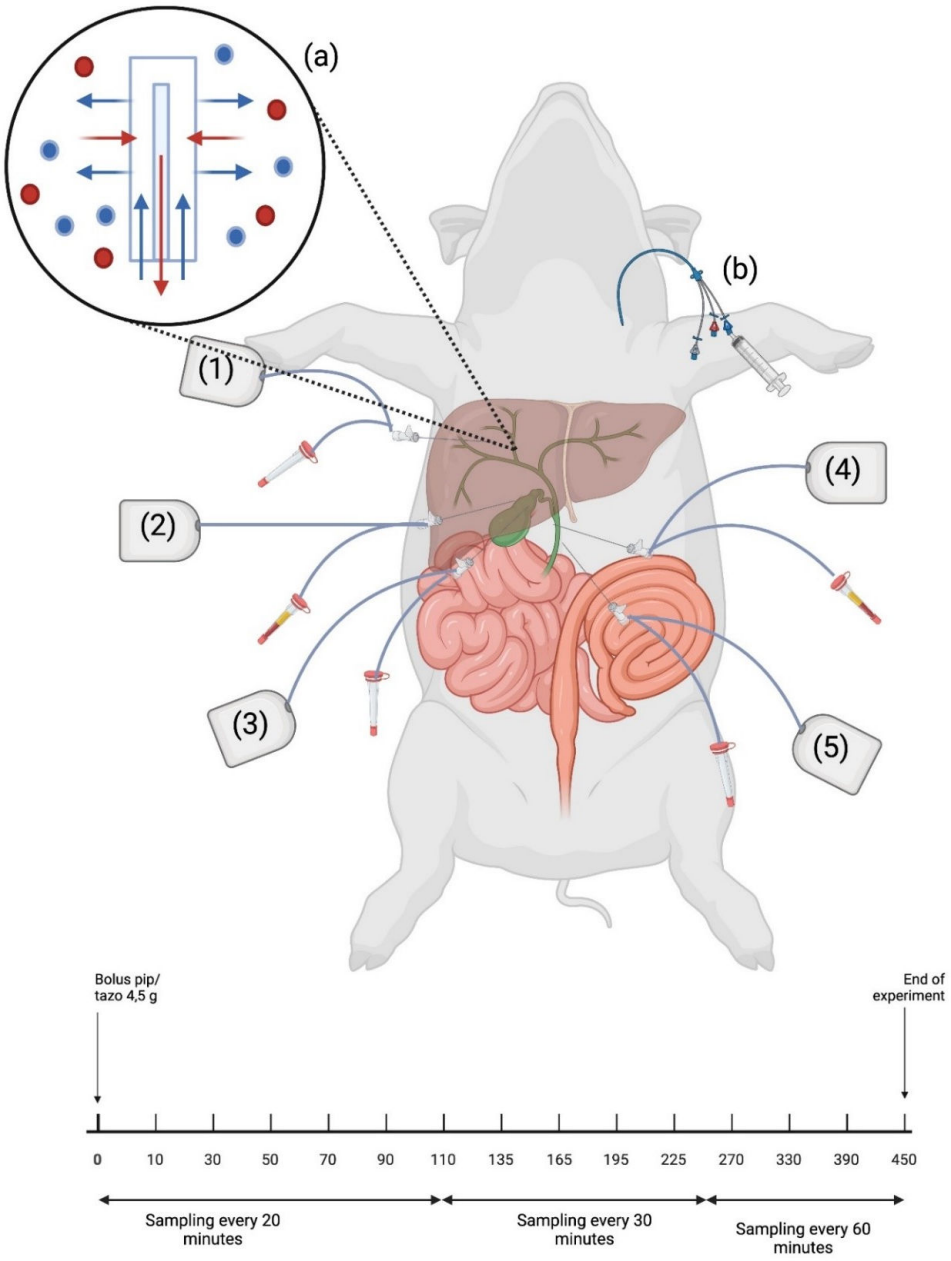
Illustrative schematic overview of microdialysis catheter placement. The catheters were placed in the liver (i), gallbladder wall (ii), gallbladder bile (iii), CBD wall (iv), and CBD bile (v). At the tip of the microdialysis catheter, a semipermeable membrane allows for diffusion of piperacillin molecules along the concentration gradient (a). For reference, venous blood samples were collected through a central venous catheter (b). From drug administration (T_0_) to 120 min, dialysates were collected at 20 min intervals, from time 120–240 min at 30 min intervals and from time 240–480 min at 60 min intervals. Figure 1 was created using BioRender with permission for publication from BioRender.

After induction of general anesthesia, a midline incision from the xiphoid process to the lower part of the abdomen was performed. Using diathermy, the fascia and peritoneum were opened with respect to the underlying intestines. The biliary system was identified, and five microdialysis catheters were placed using splitable introducers: (i) in the liver, (ii) in the gallbladder wall, (iii) in the gallbladder bile, (iv) in the common bile duct (CBD) wall, and (v) in the CBD bile (Fig. 1). The catheters were sutured to the abdominal wall to avoid catheter displacement. Finally, the abdomen was closed.

### Microdialysis

The microdialysis technique is a well-established sampling tool in pharmacokinetic studies, as it facilitates continuous sampling of the free unbound antibiotic from the extracellular fluid. The microdialysis catheters are continuously perfused, which means the concentration in the collected dialysate only represents a fraction of the absolute tissue concentration. This fraction is referred to as the relative recovery, representing a calibration factor. As such, if the absolute tissue concentration is of interest, catheter calibration is imperative. In this study, the retrodialysis by drug with an internal standard method was used (with 0.9% NaCl containing 5 mg/mL benzylpenicillin) as previously described (13).

The microdialysis system consisted of equipment from μ-Dialysis AB, Stockholm, Sweden comprising CMA 63 microdialysis catheters (membrane length: 10 mm, 20 kDa molecule cut-off) and CMA 107 precision pumps which produced a flow rate of 2 µL/min.

### Drug administration and sampling

A standard clinical dose of TZP 4/0.5 g (Fresenius, Bad Homburg, Germany) was administered as an intravenous bolus infusion over a period of 30 min. Initiation of the infusion was referred to as time 0. The dialysates were then collected at different time intervals over an 8 h period (Fig. 1). Fourteen samples were collected from each microdialysis catheter and immediately stored at −80°C until analysis. For reference, blood samples were collected from the central venous catheter in ethylenediaminetetraacetic acid tubes at the midpoint of each microdialysis sampling interval. The blood samples were stored at 5°C for a maximum of 6 h and then centrifuged at 3,000 × *g* for 10 min. Plasma was collected into Eppendorf tubes and stored at −80°C until analysis.

### UHPLC

Unbound plasma and dialysate concentrations were analyzed with an Exion-AD HPLC and Sciex 4500 qTrap MS system, as described previously (14). The lower limits of quantification for both piperacillin and benzyl-penicillin were 10 ng/mL. The mean *R*^2^ for piperacillin and benzyl-penicillin was 0.9905 and 0.9902, respectively. The coefficient of variation percentage (CV%) was below 10, calculated at concentrations of 0.07, 0.45, and 0.80 µg/mL for both drugs.

### Pharmacokinetic analysis and statistics

Microsoft Excel (Microsoft Office Standard 2016) was used to estimate the *T* > MIC for MIC 8, 16, and 32 µg/mL using linear interpolation for each compartment and pig individually. For all compartments, the mean time to reach a concentration of 8, 16, and 32 µg/mL was estimated. The standard pharmacokinetic parameters, area under the concentration-time curve (AUC_0–8h_), peak drug concentration (*C*_max_), and half-life (*T*_1/2_) were determined separately for each compartment and pig by non-compartmental analysis using the pharmacokinetic series of commands in Stata (v. 15.1, StataCorp LLC, College Station, TX, USA). AUC_0–8h_ was calculated using the linear up-log down trapezoidal rule. *C*_max_ was calculated as the maximum of all the recorded concentrations of each compartment. The *T*_1/2_ was calculated using the formula ln(2)/ λ_eq_, where λ_eq_ is the terminal elimination rate constant estimated by linear regression of the log concentration on time. The tissue AUC_0–8h_ to plasma AUC_0–8h_ ratio (AUC_tissue_/AUC_plasma_) was calculated as a measure of tissue penetration. Penetration was defined as complete when one was part of the 95% confidence interval (95% CI). All variables were analyzed using a mixed model, considering the variance between pigs. The model assumptions were tested by visual diagnosis of residuals, fitted values, and estimates of random effects. For the pharmacokinetic parameters, the assumptions were considered violated. As such, the pharmacokinetic parameters were log-transformed for statistical analyzes, and the pharmacokinetic data are given as medians (95% CI). Comparisons between compartments were assessed using a paired *t*-test. Significance was considered when *P* < 0.05. A correction for degrees of freedom due to small sample size was performed using the Kenward-Roger approximation method.

## RESULTS

All eight pigs completed the study. At autopsy, one catheter from the liver, three catheters from the gallbladder wall, two catheters from the CBD wall, and one catheter from the CBD bile were displaced and thus excluded. The mean relative recoveries (SD) were: liver 29% (12), gallbladder wall 35% (16), gallbladder bile 52% (13), CBD wall 28% (12), and CBD bile 47% (14).

### *T* > MIC and time to reach MIC

**Fig 2:**
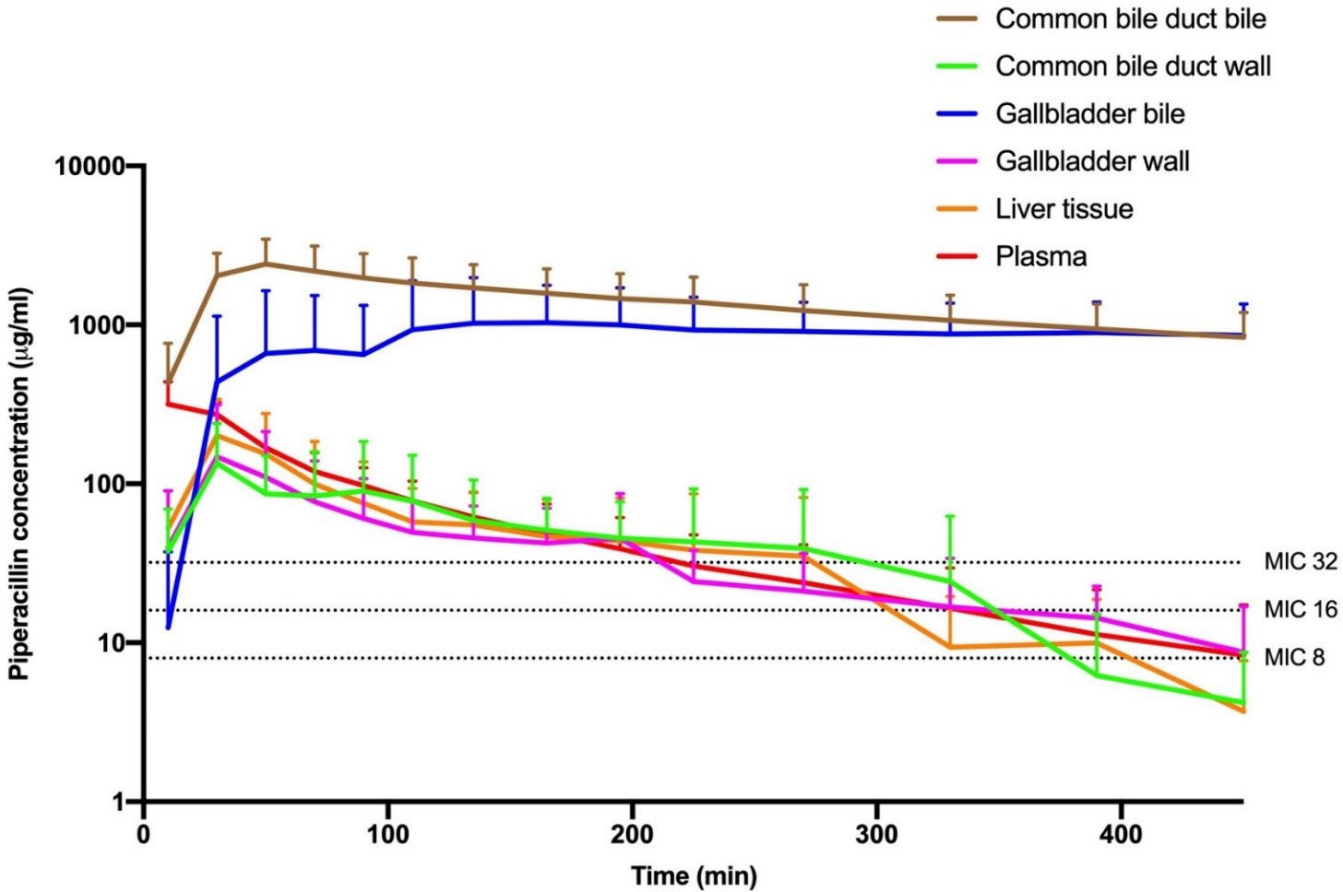
Mean concentration-time curves of piperacillin. Piperacillin concentration over time in CBD bile, CBD wall, gallbladder bile, gallbladder wall, liver, and plasma. The error bars represent standard deviations.

The mean *T* > MIC for the MIC targets of 8, 16, and 32 µg/mL along with the mean time to reach the MIC targets is displayed in Table 1, while concentration-time curves for piperacillin in the investigated compartments are presented in Fig. 2. The mean *T* > MIC (%T > MIC) varied from 345 to 446 min (77%–99%), 261–446 min (58%–99%), and 200–444 min (42%–99%) for the MIC targets of 8, 16, and 32 µg/mL, respectively.

**TABLE 1:** Time above the MIC (8, 16, and 32 µg/mL) in plasma, bile, and target tissues of the biliary system^*h*^^,^^*i*^^,^^*j*^

Parameter	*T* > MIC (min)	95%	%*T* > MIC	95%	*T* to MIC (min)	Range
MIC8						
Plasma	376	(321–431)	84	(71–96)	0	(0–1)
Liver	345	(287–404)	77	(34–90)	7	(1–37)
Gallbladder wall	400	(330–469)	89	(73–104)	16	(1–62)
Gallbladder bile	397^*a*^	(342–453)	88^*a*^	(76–101)	53	(1–148)
CBD wall	351	(288–415)	78	(64–92)	12	(1–55)
CBD bile	446^*b*^	(387–480)^*c*^	99^*b*^	(86–112)	5	(0–30)
MIC16						
Plasma	298	(240–357)	66	(53–79)	1	(0–1)
Liver	261	(198–323)	58	(44–72)	14	(1–73)
Gallbladder wall	347	(273–422)	77	(61–94)	25	(2–94)
Gallbladder bile	392^*d*^	(334–451)	87^*d*^	(74–100)	58	(2–161)
CBD wall	300	(233–368)	67	(52–82)	18	(2–82)
CBD bile	446^*e*^	(384–480)^*c*^	99^*e*^	(85–113)	5	(0–30)
MIC32						
Plasma	218	(147–289)	48	(33–64)	1	(1–2)
Liver	200	(123–276)	44	(27–61)	20	(3–95)
Gallbladder wall	187	(96–279)	42	(21–62)	33	(3–26)
Gallbladder bile	377^*f*^	(306–448)	84^*f*^	(68–100)	73	(4–172)
CBD wall	237	(154–320)	53	(34–71)	29	(4–122)
CBD bile	444^*g*^	(367–480)^*c*^	99^*g*^	(82–116)	6	(0–30)

^*a*^ Paired *t*-test of gallbladder bile compared to plasma (*P* = 0.55), liver (*P* = 0.17), gallbladder wall (*P* = 0.95), CBD wall (*P* = 0.24), and CBD bile (*P* = 0.20).

^*b*^ Paired *t*-test of CBD bile compared to plasma (*P* = 0.07), liver (*P* = 0.01), gallbladder wall (*P* = 0.29), and CBD wall (*P* = 0.03).

^*c*^ The upper limit of the 95% CI have been altered to fit the 8 h dosing interval.

^*d*^ Paired *t*-test of gallbladder bile compared to plasma (*P* = 0.02), liver (*P* = 0.00), gallbladder wall (*P* = 0.33), CBD wall (*P* = 0.04), and CBD bile (*P* = 0.20).

^*e*^ Paired *t*-test of CBD bile compared to plasma (*P* = 0.00), liver (*P* = 0.00), gallbladder wall (*P* = 0.04), and CBD wall (*P* = 0.00).

^*f*^ Paired *t*-test of gallbladder bile compared to plasma (*P* = 0.00), liver (*P* = 0.00), gallbladder wall (*P* = 0.00), CBD wall (*P* = 0.01), and CBD bile (*P* = 0.21).

^*g*^ Paired *t*-test of CBD bile compared to plasma (*P* = 0.00), liver (*P* = 0.00), gallbladder wall (*P* = 0.00), and CBD wall (*P* = 0.00).

^*h*^ All values are given as a mean.

^*i*^ *T* > MIC: time above the minimal inhibitory concentration; %*T* > MIC: percent of the time interval the concentration is above the minimal inhibitory concentration; *T* to MIC: time in minutes to MIC; MIC8 = 8 µg/mL; MIC16 = 16 µg/mL; MIC32 = 32 µg/mL.

^*j*^ CBD: Common bile duct.

No significant differences were found in the *T* > MIC between plasma and the target tissues (liver, gallbladder wall, and CBD wall) for any of the MIC targets. Similarly, no significant differences were found in the *T* > MIC between the bile compartments (gallbladder bile and CBD bile) for any of the MIC targets. However, *T* > MIC for both MIC 16 and 32 µg/mL was significantly longer for the bile compartments compared to plasma and the target tissues, except for the comparison of gallbladder bile and gallbladder wall for MIC 16 µg/mL.

A concentration of 8, 16, and 32 µg/mL was first reached in plasma within 1 min and last in gallbladder bile ranging from 53 to 73 min.

All compartments achieved the treatment target of 50%*T* > MIC for the MIC targets of 8 and 16 µg/mL. The *T* > MIC for CBD bile was 99% for all the MIC targets while gallbladder bile ranged between 84% and 88% across the MIC targets. Both bile compartments remained above the MIC targets after achievement and were therefore only limited by the penetration time in reaching the treatment target of 100%*T* > MIC.

### Pharmacokinetic parameters

The pharmacokinetic parameters AUC_0–8h_, *C*_max_, *T*_1/2_, and AUC_tissue_/AUC_plasma_ for piperacillin are displayed in Table 2.

**TABLE 2:** Piperacillin pharmacokinetic parameters for plasma, bile, and target tissues of the biliary system^*g*^^,^^*h*^^,^^*i*^

Parameter	AUC_0–8h_ (min × µg/mL)	95%	*C*_max_ (µg/mL)	95%	*T*_½_ (min)	95%	AUC_tissue_/AUC_plasma_	
Plasma	27,259	(17,177–43,259)	371	(238–579)	79	(50–124)	–	–
Liver	17,206	(10,526–28,125)	190	(120–301)	82	(49–136)	0.63	(0.33–1.22)
Gallbladder wall	13,950	(7,828–24,860)	138	(83–232)	123	(67–227)	0.5	(0.24–1.03)
Gallbladder bile	262,296^*a*^	(165,281–416,257)	1,067^*b*^	(685–1,664)	335^*c*^	(103–1,086)	9.62	(5.11–18.11)
CBD wall	17,839	(10,504–30,297)	138	(85–224)	98	(56–170)	0.63	(0.32–1.24)
CBD bile	553,821^*d*^	(338,810–905,277)	2,112^*e*^	(1,332–3,349)	600^*f*^	(359–1,004)	19.83	(10.33–38.07)

^*a*^ Paired *t*-test of gallbladder bile compared to plasma (*P* = 0.00), liver (*P* = 0.00), gallbladder wall (*P* = 0.00), CBD wall (*P* = 0.00), and CBD bile (*P* = 0.02).

^*b*^ Paired *t*-test of gallbladder bile compared to plasma (*P* = 0.00), liver (*P* = 0.00), gallbladder wall (*P* = 0.00), CBD wall (*P* = 0.00), and CBD bile (*P* = 0.00).

^*c*^ Paired *t*-test of gallbladder bile compared to plasma (*P* = 0.02), liver (*P* = 0.03), gallbladder wall (*P* = 0.13), CBD wall (*P* = 0.051), and CBD bile (*P* = 0.33).

^*d*^ Paired *t*-test of CBD bile compared to plasma (*P* = 0.00), liver (*P* = 0.00), gallbladder wall (*P* = 0.00), and CBD wall (*P* = 0.00).

^*e*^ Paired *t*-test of CBD bile compared to plasma (*P* = 0.00), liver (*P* = 0.00), gallbladder wall (*P* = 0.00), and CBD wall (*P* = 0.00).

^*f*^ Paired *t*-test of CBD bile compared to plasma (*P* = 0.00), liver (*P* = 0.00), gallbladder wall (*P* = 0.00), and CBD wall (*P* = 0.00).

^*g*^ Values are given as a median (95% CI).

^*h*^ AUC_0–8h_: area under the concentration-time curves from *t* = 0 hours to *t* = 8 hours; *C*_max_: peak drug concentration; *T*_½_: half-life; AUC_tissue_/AUC_plasma_: tissue penetration. CBD: common bile duct.

^*i*^ “–” indicates that the ratio of plasma AUC divided by plasma AUC has not been provided, as piperacillin's 100% plasma penetration makes it redundant.

AUC_0–8h_ was largest in the bile compartments and smallest in the target tissues (liver, gallbladder wall, and CBD wall). *C*_max_ was almost three to five times higher in the bile compartments compared to plasma and five to 11 times higher compared to the target tissues. Furthermore, the *T*_1/2_ in the bile compartments was significantly longer compared to plasma and the target tissues. The penetration of piperacillin was highest in the bile compartments and lowest in the target tissues.

## DISCUSSION

This is the first study to dynamically quantify piperacillin *T* > MIC in bile and target tissues of the biliary system. The concentration of piperacillin in the bile (gallbladder bile and CBD bile) was significantly higher than in plasma and the target tissues (liver, gallbladder wall, and CBD wall), with prolonged *T* > MIC for the targeted MIC values of 8, 16, and 32 µg/mL. In addition, elevated AUC and *C*_max_ as well as a prolonged *T*_1/2_ was observed in the bile compartments compared to plasma and the target tissues.

In surgical procedures involving the biliary system, surgical site infections (SSIs) present a significant challenge, with the risk varying according to the specific procedure. The risk of developing an SSI ranges from approximately 1% in laparoscopic cholecystectomies to as high as 25% in pancreaticoduodenectomies (15, 16).

To mitigate the risk of SSIs, timely and effective antibiotic prophylaxis is essential and should be administered before the procedure. Guidelines recommend that tissue concentrations exceed the MIC of the most prevalent bacterial species associated with SSIs before incision and be maintained throughout the surgical procedure (17, 18). It is suggested to administer prophylactic antibiotics 60–120 min before the procedure and re-administer after 2×T_1/2_ of the antibiotic in cases of prolonged surgery (19). However, these guidelines are based on general plasma pharmacokinetics and do not consider the PK/PD indices of specific antibiotics or the particular surgical procedure (19, 20). According to the present study, piperacillin concentrations in plasma, target tissues of the biliary system, and CBD bile reached the target MIC of 16 µg/mL after a mean of 28 min, while gallbladder bile concentrations reached the target after a mean of 58 min. These concentrations remain above the MIC 16 µg/mL for at least 261 min (4.4 h). This suggests that TZP should be administered approximately 60 min before a surgical procedure involving the biliary system, with re-administration necessary after 4.4 h.

Achieving high concentrations of piperacillin and prolonged *T* > MIC and *T*_1/2_ in bile appears beneficial for treating biliary system infections such as cholecystitis and cholangitis. However, since most bacteria inhabit the extracellular fluid of tissues, it is essential also to attain therapeutic concentrations within the tissues of the biliary system. The treatment target of 50%*T* > MIC of 8 and 16 µg/mL was reached in all the compartments, but only gallbladder and CBD bile approached the more aggressive treatment targets of 100%*T* > MIC and 100%T > 4× MIC, only limited by the penetration time. Thus, steady-state concentrations may approach 100%*T* > MIC for the MIC targets of 8, 16, and 32 µg/mL. To enhance target attainment in the other compartments, increased dosing or alternative administration forms (prolonged or continuous) should be considered. However, higher dosages may lead to elevated peak concentrations, which are not always advantageous, as high plasma concentrations have been associated with increased neurotoxicity, nephrotoxicity, and even higher mortality (21, 22). Conversely, prolonged and continuous infusion has shown promise in achieving longer *T* > MIC without reaching toxic concentrations (23, 24). Whether achieving the treatment targets of 100%*T* > MIC and 4× MIC in bile and the target tissues would further improve treatment outcomes remains to be investigated.

Continuous infusion may also be advantageous for treating serious infections caused by *Pseudomonas aeruginosa* or *Enterobacterales* with intermediate susceptibility towards TZP. The standard treatment for these infections is TZP 4.0/0.5 g every 6 h. In our study, the *T* > MIC has been estimated based on an 8 h dosing interval. Across compartments, the lowest mean *T* > MIC was observed in the liver, with a mean *T* > MIC of 261 min (4.35 h) for MIC 16 µg/mL, corresponding to 83%*T* > MIC 16 during a 6 h dosing interval. Whether this is sufficient for this group of bacteria remains to be investigated.

Few studies have examined piperacillin concentrations within the biliary system (6, 7, 25–28). Comparing our results with the results from these studies is challenging due to varying piperacillin dosages (1–5 g) and routes of administration (intramuscular or intravenous) (6, 7, 25–28). Additionally, the majority of these studies were, to the best of our knowledge, conducted before the establishment of PK/PD indices, why *T* > MIC in the biliary system has not been previously described. Despite these differences, the studies consistently report a trend of higher piperacillin concentrations in bile compared to plasma (6, 7, 25, 26). Furthermore, the concentration in CBD bile seems to be higher than that in gallbladder bile, regardless of the administered dosage. These trends align with the findings of our study.

Microdialysis has previously been used to assess piperacillin tissue concentrations in various clinical contexts. However, these studies are heterogeneous in design, with differences in patient populations, microdialysis methodological setup, tissue types, sampling periods, and administration routes, with resulting variations in reported relative recoveries (29–33). This variability highlights the challenges of making direct comparisons across study design.

Our study has several limitations that warrant discussion. First, it was conducted in pigs. Although pigs have anatomical and physiological similarities to humans, the primary elimination route of piperacillin in pigs is unknown. In humans, up to 60% of piperacillin is excreted in urine (34). We did not measure urine concentrations and, therefore, cannot evaluate whether this applies to pigs. However, a proportion of piperacillin appears to be excreted via the bile, a finding consistent with human data (7). Second, the piperacillin concentrations were determined in samples from healthy tissue. The presence of an infection, cancer, or a surgical procedure leads to inflammation, potentially altering the drug delivery. Third, although high concentrations of piperacillin were achieved in bile, we did not assess the antimicrobial activity in this matrix. Human bile has been shown to reduce the efficacy of several antibiotics compared to standard growth media, suggesting that high concentrations alone may not guarantee sufficient bacterial killing (35). In addition, bile may promote antimicrobial resistance in some bacterial species (36). Fourth, although piperacillin is co-administered with tazobactam to counteract β-lactamase-producing organisms, we only quantified piperacillin concentrations. This limits our ability to fully assess the antimicrobial coverage, particularly against β-lactamase-producing bacterial pathogens and anaerobes. Further, this model does not encounter varying degrees of biliary tract obstruction. Whether obstruction and stasis of the biliary system affect the piperacillin concentrations in bile and biliary tract tissue should be investigated in future studies. Finally, we only collected samples during a single 8 h dosing interval, which did not allow piperacillin to reach steady-state conditions. Therefore, our results cannot readily be extrapolated to a therapeutic setting and are more representative of prophylactic use in surgical procedures.

### Conclusion

Piperacillin demonstrated a significant ability to distribute to bile, achieving high peak drug concentrations and prolonged excretion, resulting in prolonged *T* > MIC in bile compared to plasma and target tissues in the biliary system. Administering TZP for prophylaxis and treatment for surgical procedures and infections involving the biliary system therefore seems favorable. For surgical prophylaxis, to attain a MIC target of 16 µg/mL, piperacillin should be administered approximately 60 min before the procedure and re-administered after 4 h. When it comes to the treatment of infection, piperacillin concentrations in bile and the target tissues achieved the treatment target of 50%*T* > MIC for MIC 8 and MIC 16 µg/mL. To achieve the treatment targets of 100%*T* > MIC and 100%*T* > 4× MIC, prolonged or continuous infusion may be necessary for increased treatment success; this, however, needs further investigation in future studies.
